# A Chinese Herbal Preparation Containing Radix Salviae Miltiorrhizae, Radix Notoginseng and Borneolum Syntheticum Reduces Circulating Adhesion Molecules

**DOI:** 10.1093/ecam/nen060

**Published:** 2011-06-23

**Authors:** Kylie A. O'Brien, Shanhong Ling, Estelle Abbas, Aozhi Dai, Jiansheng Zhang, Wen Cheng Wang, Alan Bensoussan, Ruizhi Luo, Zhi-Xin Guo, Paul A. Komesaroff

**Affiliations:** ^1^Monash University Department of Medicine, Alfred Hospital, Melbourne, Australia; ^2^Faculty of Health, Engineering and Science, Victoria University, P.O. Box 14428, Melbourne, VIC 8001, Australia; ^3^Division of Chinese Medicine, School of Health Sciences, RMIT University, Melbourne, Australia; ^4^Registered Chinese medicine practitioner in private practice, Prahran, VIC, Australia; ^5^National Institute of Complementary Medicine, University of Western Sydney, Sydney, Australia; ^6^Institute of Research and Development, Tianjin Tasly Pharmaceutical Corporation, Tianjin, China

## Abstract

Circulating adhesion molecules (CAMs), surface proteins expressed in the vascular endothelium, have emerged as risk factors for cardiovascular disease (CVD). CAMs are involved in intercellular communication that are believed to play a role in atherosclerosis. A Chinese medicine, the “Dantonic Pill” (DP) (also known as the “Cardiotonic Pill”), containing three Chinese herbal material medica, Radix Salviae Miltiorrhizae, Radix Notoginseng and Borneolum Syntheticum, has been used in China for the prevention and management of CVD. Previous laboratory and animal studies have suggested that this preparation reduces both atherogenesis and adhesion molecule expression. A parallel double blind randomized placebo-controlled study was conducted to assess the effects of the DP on three species of CAM (intercellular cell adhesion molecule-1 (ICAM-1), vascular cell adhesion molecule-1 and endothelial cell selectin (E-selectin)) in participants with mild-moderate hypercholesterolemia. Secondary endpoints included biochemical and hematological variables and clinical effects. Forty participants were randomized to either treatment or control for 12 weeks. Treatment with DP was associated with a statistically significant decrease in ICAM-1 (9% decrease, *P* = .03) and E-Selectin (15% decrease, *P* = .004). There was no significant change in renal function tests, liver function tests, glucose, lipids or C-reactive protein levels and clinical adverse effects did not differ between the active and the control groups. There were no relevant changes in participants receiving placebo. These results suggest that this herbal medicine may contribute to the development of a novel approach to cardiovascular risk reduction.

## 1. Introduction

Circulating adhesion molecules (CAMs) are proteins expressed by the vascular endothelium involved in intercellular communication that are believed to play a role in the initiation of the atherosclerotic process. There are several families of CAMs, including integrins, cadherins, selectins and immunoglobulin superfamily members [1]. The most important adhesion molecules involved in atherosclerosis appear to be intercellular cell adhesion molecule-1 (ICAM-1), endothelial cell selectin (E-selectin) and vascular cell adhesion molecule-1 (VCAM-1). Raised levels of ICAM-1 have been found to be predictive of increased risk of cardiovascular events [2, 3], including in healthy individuals [4]. Levels of P-selectin and E-selectin have been found to predict cardiovascular disease (CVD) risk in healthy women [5], and in patients with stable coronary artery disease plasma levels of E-selectin appear to reflect the severity of systemic atherosclerosis [6]. VCAM-1 may be a more specific marker for advanced atherosclerosis, since it is usually expressed in atherosclerotic plaques [7] and higher levels are associated with increased risk of coronary events in persons with existing CVD [2]. There is some evidence that measures, which reduce circulating levels of adhesion molecules also reduce cardiovascular risk [8, 9].

The use of herbs and herbal therapies in Western societies is extensive and increasing, especially for the prevention of illness and the management of chronic disease. Radix Salviae Miltiorrhizae (Chinese herb Dan Shen) and Radix Notoginseng (San Qi) are medicinal herbs (material medica) commonly used in traditional Chinese medicine practice to prevent or treat conditions relating to the heart and blood vessels. A mixture of these two herbs, together with a third materia medica—Borneolum Syntheticum (Bing Pian)—is produced by the Tianjin Tasly Pharmaceutical Co., Ltd, Tianjin, China, under the name of the “Dantonic Pill" (DP) (also known as the “Cardiotonic Pill"), under conditions of “Good Manufacturing Practice" (GMP) and “Good Laboratory Practice" (GLP) verified by Chinese, Australian and US Government agencies. This product has been sold in China for ~8 years, predominantly for the prevention and treatment of CVD [10]. Laboratory studies have shown that this product displays a range of activities in the cardiovascular system, including reduction in venous thrombosis [11] and blood viscosity [12].

We have previously shown that in cultured human vascular endothelial cells DP significantly attenuates tumor necrosis factor *α*-induced expression of adhesion molecules ICAM-1 and VCAM-1 in a dose-dependent manner and inhibits platelet-derived growth factor BB-induced DNA synthesis and cell proliferation in cultured human vascular smooth muscle cells [13, 14]. In addition, we have found that in a mouse model of atherosclerosis—apolipoprotein E deficient mice fed an atherogenic diet—administration of DP prevents increases in VCAM-1 expression and inhibits the development of fatty streaks and atherosclerotic plaques in the thoracic aorta without altering levels of circulating lipids [15].

The study reported here was designed to examine the effects of DP on expression of adhesion molecules in human subjects with mild to moderate hypercholesterolemia and to obtain data about the tolerability and safety of this product in a Western population.

## 2. Methods

### 2.1. Study Design and Participants

The study employed a parallel double-blinded, randomized, placebo controlled design. Forty subjects with mild to moderately elevated cholesterol were randomly allocated to receive either DP or matching placebo for 12 weeks. The study was part of a larger investigation of the effects of Chinese medicinal herbs on cardiovascular risk, other aspects of which will be reported elsewhere. The study was carried out in accordance with the Declaration of Helsinki (2000) of the World Medical Association.

Participants were healthy individuals aged 18 years and over with a total plasma cholesterol of 5.0 mmol/l or above, triglyceride level of >2 mmol/l or HDL level of <1.0 mmol/l. Exclusion criteria included plasma triglyceride levels over 10 mmol/l, established heart disease, other serious medical or psychological conditions, pregnancy, current use of vasoactive medications, warfarin and postmenopausal hormone therapy. Participants taking omega-3 fish oil supplements were asked to cease these at least 3 weeks prior to entering the study. Participants were recruited via newspaper advertisements and posters displayed at the Alfred Hospital in Melbourne, Australia. Ethics approval was obtained from the Alfred Hospital Ethics Committee and Monash University and all participants gave full consent.

Following baseline assessment (Visit 1) participants were randomized to receive either DP or placebo in capsule form, three capsules three times per day for 12 weeks, after which the assessment was repeated (Visit 2). Participants were asked to maintain their current diets and exercise regimes during the study period. They were contacted by telephone every 3 weeks to ascertain side effects and encourage compliance, which was assessed by examination of unused returned packets. They were also asked to complete a 4-day diet diary and exercise questionnaire during the first and last weeks of the study.

### 2.2. Outcome Variables

The primary outcome variables were circulating levels of ICAM-1, VCAM-1 and E-selectin. Secondary outcomes included serum urea, electrolytes, creatinine, glucose, liver function, lipids, full blood examination and high-sensitivity C-reactive protein.

### 2.3. Chinese Herbal Mixture

The DP is an herbal product produced by Tianjin Tasly Pharmaceutical Corporation Ltd in China consisting of a mixture of three Chinese materia medica: Radix Salviae Miltiorrhizae, Radix Notoginseng and Borneolum Syntheticum. Both the preparation and the individual herbs have been approved by the Australian Therapeutic Goods Administration for use in Australia and are widely available.

Animal toxicity studies of the DP conducted in China and reported by the Tianjin Tasly Pharmaceutical Company demonstrated no toxic effects in mice in studies up to 6 months duration. The lethal dosage (LD50) of DP extract was assessed via single gastric canal on mice and was calculated using the “Bliss Method" as recommended in the Chinese Government's *New Drug (Chinese Medicine) Pre-Clinical Research Guidelines* [16]. The LD50 was found to be 25.807 g/kg, equivalent to 3934 times the intended clinical human oral dose of 6.56 mg/kg.

### 2.4. Laboratory Techniques

Expression of ICAM-1, VCAM-1 and E-selectin in circulating leukocytes was determined by fluorescence-activated cell sorting (FACS) analysis. Whole blood cells were collected after separation of the plasma, incubated in the lysis buffer (BD Pharm Lyse (Cat. No. 555899), BD Biosciences Pharmingen, San Diego, CA, USA) for 10 min at 37°C and washed in PBS to remove the red blood cells, and the leukocytes were re-suspended in 0.5 mL Hanks' balanced salt solution (HBSS). Cells were incubated with a specific fluorescence-connected antibody against ICAM-1, VCAM-1 or E-selectin (BD Biosciences, San Diego, CA, USA) for 1 h, protected from light, then washed in 0.5 mL HBSS to remove residual antibody and analyzed using the FACS scanning flow cytometer and CellQuest software (Becton Dickinson, Franklin Lakes, NJ, USA).

Assays for other serum markers were conducted in the Pathology Department of the Alfred Hospital using established techniques. These included total serum cholesterol, triglycerides, HDL-cholesterol and C-reactive protein (Roche method for first 3 months then Abbott method for remainder of study, cross-calibrated for consistency of values), LDL cholesterol (Abbott, Illinois, USA), homocysteine (Abbott AxSym), fibrinogen (Von Claus method on Diagnostica Astago STAR), Von Willebrand Factor (ELISA manual method).

### 2.5. Statistical Analysis

The study was designed to be able to identify a 10% change in total serum cholesterol and LDL-cholesterol with a Type 1 error of 0.05 with a power of 0.8. Within-groups analyses were conducted on all defined outcome variables. Where data were normal, the paired Student *t*-test was used. Where tests for normality of data were violated the Wilcoxon Signed Ranks Test was used. A two-sided *P*-value of .05 was considered as statistically significant.

## 3. Results

A total of 40 subjects, 20 in each medication group, completed the study protocol. The characteristics of participants in each group are set out in Table 1. There was no significant difference between the two groups in terms of mean age of participants, alcohol use, physical activity, dietary content or body mass index. Physical activity did not change significantly in either group over the study period, as measured by the Baecke Physical Activity Questionnaire [17].

### 3.1. CAM

Circulating levels of ICAM-1 and E-selectin decreased significantly in the DP group (by 9 and 15%, resp.). In the Placebo group there were no significant changes in any of the CAMs tested. There was no significant change in VCAM-1 levels in either group (Table 2).

### 3.2. Adverse Effects

There was no difference in reported adverse effects between the two groups. Five participants in the Placebo group and three in the DP group reported various symptoms, predominantly gastrointestinal in nature, such as bloating, alteration of stools, mild nausea, belching and gastric reflux. One participant in the Placebo group discontinued the study 6 weeks after randomization due to indigestion.

### 3.3. Biochemical and Hematological Analyses

There were no clinically or statistically significant changes in urea and electrolytes, blood glucose, lipids, hemoglobin, platelet count, white cell count or C-reactive protein in either group (data not shown). There were minor changes in derived hematological parameters and bilirubin within the reference range for normal adults in both groups, which were not different between the two groups. The liver enzyme alanine transferase declined significantly within the reference range in the DP group (*P* = .02) but did not change in the Placebo group.

## 4. Discussion

This study has shown that 12 weeks administration of the Chinese herbal mixture DP to men and women with mild-to-moderate elevation in lipid levels is associated with reduced expression of the CAMs ICAM-1 and E-selectin. The study has also shown that in this setting the preparation is well tolerated and free from significant biochemical adverse effects.

CAMs have emerged as a risk factor for CVD. They are believed to play important roles in the initiation of the atherosclerotic process by mediating various steps in leukocyte recruitment. Upregulation of adhesion molecule expression in the development of atherogenesis has been observed in both animal models and humans [18, 19]. Leukocyte recruitment to atherosclerosis sites is mediated by CAM expressed on the surface of endothelial cells, which bind circulating leukocytes to the vascular endothelium in the early phase of atherosclerosis [6]. CAM gene expression is upregulated in inflammatory states through cytokine release by various cells including endothelial cells, smooth muscle cells and leukocytes [1]. Hemodynamic shear stress is also able to increase ICAM-1 expression in addition to cytokine stimulation [20]. ICAM-1 and VCAM-1 mediate stronger attachment of leukocytes to the endothelium whilst the selectins (E-selectin, P-selectin) mediate transient leukocyte rolling on the endothelial surface [7, 20].

Raised levels of ICAM-1 have been found to be predictive of increased risk of cardiovascular events [2–4]. In patients with stable coronary artery disease plasma levels of E-selectin is synthesized and exported on the endothelial cell surface in inflammatory situations [21] and appears to reflect the severity of systemic atherosclerosis [6]. P-selectin levels predict CVD risk in healthy women [5]. VCAM-1 may be a specific marker for advanced atherosclerosis, since it is usually expressed on atherosclerotic plaques [7] and VCAM-1 levels correlated with extent of atherosclerosis [22]. In view of these facts, it has been argued that adhesion molecules and their interactions may be useful therapeutic targets for the reduction of cardiovascular risk and management of CVD [23, 24].

These CAMs are produced chiefly by endothelial cells, although modest expression of ICAM-1 and VCAM-1 has also been shown by bone marrow stromal cells and some other cells under specific conditions [25]. Circulating levels of CAMs increase leukocyte binding that can be accurately quantified by FACScan analysis [1, 2]. Our results show clearly that ICAM-1, VCAM-1 and E-selectin are detectable on the surface of leukocytes and that treatment with DP for 12 weeks reduces expression of E-selectin and ICAM-1.

Several effects of Radix Salviae Miltiorrhizae on the cardiovascular system have been demonstrated, including reduction of cholesterol and triglyceride levels and enhanced recovery following myocardial ischemia [26, 27] and reduced adhesion molecule expression [28, 29]. Radix Salviae Miltiorrhizae contains multiple active chemical constituents, of which nearly 100 have been identified. Among the most important of these are lipophilic tanshinones such as tanshinone IIA and hydrophilic phenolic acids, particularly danshensu, protocatechuic aldehyde and salvianolic acid [30].

Radix Notoginseng has been used traditionally for its haemostatic effect for internal and external bleeding [31], is said to increase coronary blood flow and reduce myocardial oxygen consumption [32] and dilate coronary arteries [26]. The active constituents of Radix Notoginseng include arasaponin A, arasaponin B, dencichine and trilinolein [31]. Saponins have been found to lower blood lipids [33], limit atherosclerosis by inhibiting the proliferation of aortic smooth muscle cells [34, 35] and reduce inflammation [36]. Trilinolein has antioxidant effects, reduces thrombogenicity and suppresses cardiac arrhythmias during oxygen-deprivation ischemia in a rat model [37]. The major chemical constituents of Borneoleum Syntheticum are sesquiterpenes: racemic borneol, isoborneol and camphor [38].

In the present study, the effect of the mixture of herbs contained in the DP appears specifically to reduce circulating levels of the adhesion molecules ICAM-1 and E-selectin. Taken together with the effect previously demonstrated in an animal model of reduced atherogenesis this suggests that this product may provide a useful model for the further investigation of the underlying mechanisms of CVD. It is also possible that it may also contribute to the development of novel strategies for preventing CVD and limiting the development of existing atherosclerotic lesions. Further research is necessary to identify the constituents of this herbal preparation, which are responsible for these actions.

It is important, however, to note the limitations of this study, which comprised small numbers, focused on a population of basically healthy individuals not taking conventional medications and relied on surrogate rather than clinical endpoints. It is possible that the effects of the herbal product are different in other clinical settings and in particular, in subjects with active CVD. The significance of changes in circulating adhesions molecules of this magnitude is uncertain. It is known that physical exercise can reduce adhesion molecule expression while psychological stress can increase it [39]. In our study physical exercise did not change. While there is no reason to believe that there were differences in levels of psychological stress between the groups nor changes in stress levels over the study period, we did not measure these directly so stress should be recognized as a potential confounding factor.

In summary, in human subjects with mild to moderately elevated lipid levels the mixture of herbs contained in the DP appears to have a specific effect on reducing the CAMs ICAM-1 and E-selectin. This product may contribute to the further investigation of the mechanisms of CVD and to the development of novel strategies for both its prevention and management.

## Figures and Tables

**Table 1:** Baseline characteristics of experimental and control groups.

Characteristic	DP group (*n* = 20)	Placebo group (*n* = 20)
Male/female	8/12	7/13
Mean age (years)	53.3 ± 10.7	51.6 ± 14.4
Body mass index (kg/m^2^)	26.0 ± 4.4	25.2 ± 3.6^a^
Smokers	0^a^	4
Mean no. standard alcoholic drinks/week	3.6 ± 2.6^a^	4.7 ± 4.1

^a^ *n* = 19 (data not shown for one subject).

**Table 2:** Within-groups analysis: CAMs (ICAM 1, VCAM and E-selectin).

		Visit 1	Visit 2	Visit 1	Visit 2	Visit 1	Visit 2
		ICAM-1	ICAM-1	VCAM-1	VCAM-1	E-SEL	E-SEL
DP	Mean	37.1 ± 26.1	33.8 ± 25.8	14.2 ± 10.1	14.0 ± 9.9	33.0 ± 25.3	28.0 ± 25.7
	*P* (two-tailed *t*-test)		0.03		0.79		0.004^a^
	*N* = 19						
Placebo	Mean	30.1 ± 20.6	27.4 ± 21.2	12.0 ± 8.1	11.5 ± 6.8	27.9 ± 16.2	24.8 ± 19.1
	*P* (two-tailed *t*-test)		0.36^a^		0.97^a^		0.37^a^
	*N* = 20						

E-SEL = E-selectin; ^a^Wilcoxon Signed Ranks Test; Visit 1—baseline measurements; Visit 2—measurements taken after 12 weeks of study medication.
